# Gut Microbiota Modulation and Its Relationship with Obesity Using Prebiotic Fibers and Probiotics: A Review

**DOI:** 10.3389/fmicb.2017.00563

**Published:** 2017-04-04

**Authors:** Dinesh K. Dahiya, Renuka, Monica Puniya, Umesh K. Shandilya, Tejpal Dhewa, Nikhil Kumar, Sanjeev Kumar, Anil K. Puniya, Pratyoosh Shukla

**Affiliations:** ^1^Advanced Milk Testing Research Laboratory, Post Graduate Institute of Veterinary Education and Research – Rajasthan University of Veterinary and Animal Sciences at BikanerJaipur, India; ^2^Department of Biochemistry, Basic Medical Science, South Campus, Panjab UniversityChandigarh, India; ^3^Food Safety Management System Division, Food Safety and Standards Authority of IndiaNew Delhi, India; ^4^Animal Biotechnology Division, National Bureau of Animal Genetic ResourcesKarnal, India; ^5^Department of Nutrition Biology, Central University of HaryanaMahendergarh, India; ^6^Department of Life Sciences, Shri Venkateshwara UniversityJP Nagar, India; ^7^Department of Life Science, Central Assam UniversitySilchar, India; ^8^College of Dairy Science and Technology, Guru Angad Dev Veterinary and Animal Sciences UniversityLudhiana, India; ^9^Dairy Microbiology Division, ICAR-National Dairy Research InstituteKarnal, India; ^10^Enzyme Technology and Protein Bioinformatics Laboratory, Department of Microbiology, Maharshi Dayanand UniversityRohtak, India

**Keywords:** gut microbiota, prebiotic, probiotics, obesity, nanotechnology

## Abstract

In the present world scenario, obesity has almost attained the level of a pandemic and is progressing at a rapid rate. This disease is the mother of all other metabolic disorders, which apart from placing an added financial burden on the concerned patient also has a negative impact on his/her well-being and health in the society. Among the various plausible factors for the development of obesity, the role of gut microbiota is very crucial. In general, the gut of an individual is inhabited by trillions of microbes that play a significant role in host energy homeostasis by their symbiotic interactions. Dysbiosis in gut microbiota causes disequilibrium in energy homeostasis that ultimately leads to obesity. Numerous mechanisms have been reported by which gut microbiota induces obesity in experimental models. However, which microbial community is directly linked to obesity is still unknown due to the complex nature of gut microbiota. Prebiotics and probiotics are the safer and effective dietary substances available, which can therapeutically alter the gut microbiota of the host. In this review, an effort was made to discuss the current mechanisms through which gut microbiota interacts with host energy metabolism in the context of obesity. Further, the therapeutic approaches (prebiotics/probiotics) that helped in positively altering the gut microbiota were discussed by taking experimental evidence from animal and human studies. In the closing statement, the challenges and future tasks within the field were discussed.

## Introduction

Obesity is a pathological state marked by the accumulation of excess body mass in the abdominal region as a result of disequilibrium between energy intake and its consumption. It is a metabolic disorder that is on the rise globally and if allowed to spread unchecked would assume the proportions of a pandemic. Obesity is the mother of many other deadly diseases, particularly diabetes, cardiovascular, non-alcoholic fatty liver disease (NAFLD) and some form of cancers (Kopelman, 2007; Nikolopoulou and Kadoglou, 2012; Vucenik and Stains, 2012). Obesity not only affects the well-being of a person, but also places an unwanted economic burden on the society (Wang et al., 2011; Withrow and Alter, 2011). According to a report, more than 500 million people across the world are living with the stigma of obesity, that shows the severity of the disease and the challenges confronting health practitioners (Swinburn et al., 2011). Several factors such as host genetics, metabolism, lifestyle, and diet have been pinpointed as the key etiological agents responsible for the progression of obesity. However, the in-depth mechanisms that lead to the development of obesity are yet to be disclosed. The most recent studies have speculated that the gut microbiota present in the human gastrointestinal tract (GIT) have a paramount role in the onset and establishment of obesity. The adhered gut microbiota affects the host’s nutrients acquisition and energy homeostasis by influencing the number of effector molecules that finally decide the fat storage in adipocytes (Rosenbaum et al., 2015). Nonetheless, there is growing evidence that some dietary substances, especially probiotics and prebiotics can modulate the gut microbiota of the host in a positive way and are therefore considered as important assets in the management of obesity. Various approaches such as omics methods, systems biology and metabolic engineering enable us to understand and optimize the metabolic processes (Yadav et al., 2016a,b). The major objectives of this review are to provide an overview of how prebiotics and probiotics modulate the gut microbiota in context of prevention or treatment of obesity. Before we progress further, we elaborate our current understanding of how gut microbiota are predisposed toward obesity.

## Relationship Between “Gut Microbiota and Obesity”

### Human Gut Microbiome, the “Unforeseen Organ”

It is believed that the gut of a fetus during the intrauterine period is deprived of any bacterial communities, i.e., it is nearly sterile; however, some microbes before birth and during parturition transit from the mother to the fetus gut and constitute the rudimentary microbiota (Aagaard et al., 2014). The gut composition of a child varies widely during the first few years of life due to factors like changes in gut physiology, introduction of solid foods, use of therapeutic drugs, host genotype and proximity to adult microbiota (Koenig et al., 2011). During adolescence, however, the gut microbiota is nearly consistent and predominated by a few colonizers. Thereafter, it changes during old age when the host physiology and dietary habits change dramatically. Nevertheless, the dynamics and structure of an individual’s gut microbiota is unique and people can actually be identified on the basis of microbiota “fingerprints” alone, with the help of the metagenomics approach (Franzosa et al., 2015). The gut harbors a trillion microbes, thereby constituting a complex microbial community that is approximately comprised of 1000–1100 different bacterial species altogether representing 10^14^–10^15^ microbes. This population is 10 times the number of cells present in a eukaryotic host (Qin et al., 2010) and resemble a “world within a world.” The collective genes of these different microbial species are termed as “microbiome,” while a combination of microbiome and host genes is called “metagenome” (Quigley, 2011). Before the advent of sophisticated sequencing techniques, the gut remained a neglected organ because of the limitations of culturing methods, but it is now considered to be a vital organ as it helps in various metabolic functions of the host that would otherwise not be possible (Sommer and Bäckhed, 2013). An earlier study inferred that the gut of an adult human being is mainly inhabited by bacteria from three major divisions, the *Firmicutes* (Gram-positive), *Bacteroidetes* (Gram-negative) and *Actinobacteria* (Gram-positive), which together make up more than 90% of total bacteria presented in the gut. In case of Archaea, one species *Methanobrevibacter smithii* predominates over others (Eckburg et al., 2005). However, obtaining an accurate picture of the gut is very difficult as several factors such as availability of oxygen, diet, and physiochemical properties of the gut (e.g., pH, bile) rapidly influence its composition.

Arumugam et al. (2011) made an attempt to understand the variation in species composition and gene pools within the human population from the previously available data and found the existence of three main distinct bacterial communities or “enterotypes” – *Bacteroides*, *Prevotella*, and *Ruminococcus-*based on their abundance (Arumugam et al., 2011). Later studies reduced the concept of three enterotypes to two – *Bacteroides* and *Prevotella* (Koren et al., 2013; Knights et al., 2014). From above studies, it can be inferred that gut microbiota have occupied a significant position in human biology that interplays with the metabolic physiology and influences the health status.

### Evidence that Gut Microbiota Have a Role in Obesity and Dysbiosis

The pioneering evidence that linked gut microbiota to the development of obesity came from the findings of Bäckhed et al. (2004), when they transplanted the microbiota from normally grown mice to germ free (GF) mice. The latter, consequently, gained more fat pad mass and body weight despite reduction in food consumption. Increased body weight led to insulin resistance, along with higher glucose and leptin levels in blood. The authors postulated that the transplanted microbiota helped GF mice in harvesting excess energy from the diet. Further, they advocated that microbiota increases the expression of key transcriptional factors to enhance lipogenesis in the liver and promoted lipoprotein lipase (LPL) activity to store triglyceride (TG) in adipocytes (Bäckhed et al., 2004). Surprisingly, when GF mice were maintained on a high fat diet (HFD), they were protected from the development of obesity. Interesting evidence in this context emerged from the effect of antibiotic experiments on body weight. Antibiotics have been used in the livestock sector for decades to promote the growth and body weight of animals, which indirectly indicate that role of the gut microbiota in weight modulation. Evidence from mice has shown that early exposure to antibiotics had altered their gut microbiota, increased fat mass, and negatively modulated hepatic metabolism and associated hormones, which predisposed them toward adiposity (Cho et al., 2012; Cox et al., 2014). The effect of early administration of antibiotics on human adiposity has also been seriously reviewed over the past few years (Mueller et al., 2014; Turta and Rautava, 2016; Podolsky, 2017) and there is growing consensus that their increased use maybe a reason for the obesity explosion we are witnessing today.

If microbiota have a crucial role in the development of obesity, then it is obvious that the obese phenotype should have a microbial composition distinct from lean individuals. Ley et al. (2005) during the analysis of the gut microbiota from *ob*/*ob* mice, lean *ob*/*+* and wild-type counterparts, found that genetically obese mice have more of *Firmicutes* and less of *Bacteroidetes* compared with lean mice (Ley et al., 2005). These *Firmicutes* help the obese mice to draw more calories from the ingested diet, leading to obesity (Turnbaugh et al., 2006). Upon transplantation of microbiota from obese mice to GF mice, the obese phenotype is transferred. Similar findings were observed with obese people who had less of *Bacteroidetes* and more of *Firmicutes* in their gut. The proportion of *Bacteroidetes* increased with the initiation of a low calorie diet (Ley et al., 2006). In another study, obese children were found to have more of *Firmicutes* and less of *Bacteroidetes* in their gut. In fact, they also had higher short chain fatty acids (SCFAs) that were correlated with the development of obesity (Riva et al., 2017). Overall, obese people have less microbial diversity in comparison with lean ones (Le Chatelier et al., 2013) and dietary intervention may improve the microbial richness and associated clinical phenotypes (Cotillard et al., 2013).

Alterations in the gut microbial population also occurred at genus and species level, but these results were not consistent, especially in case of *lactobacilli*. In some findings, increase in the population of *lactobacilli* was observed in obese subjects and correlated with its pro-obesity effects (Armougom et al., 2009; Million et al., 2012b). In contrast, several studies have documented their anti-obesity effects as discussed elsewhere in a review (Arora et al., 2013). This mystery was resolved with the help of a meta-analysis study which depicted that anti-obesity activity of *lactobacilli* is species-specific attribute and is not a common feature of whole genera (Million et al., 2012a). Likewise, the population of *bifidobacteria* is negatively correlated with obesity, and its supplementation provided anti-obesity effects in some findings (Yin et al., 2010; An et al., 2011). In addition, *Faecalibacterium prausnitzii* and *Akkermansia muciniphila* were also found to be significantly linked with obesity. In general, *F. prausnitzii* found abundant in healthy adults and its supplementation in mice have colitis preventive effects (Miquel et al., 2013). However, there is inconsistency in *F. prausnitzii* population among obese human subjects. As in one case study their population was found to be increased in obese subjects (Balamurugan et al., 2010) while in a recent finding, opposite results were obtained (Dao et al., 2016). Whereas, Feng et al. (2014) in reported non-significant results in their findings. Similarly, *A*. *muciniphila* is negatively correlated with obesity (Schneeberger et al., 2015; Remely et al., 2016) and its administration has weight lowering effects (Everard et al., 2013; Dao et al., 2016).

The above findings clearly indicate that gut microbiota have a crucial role in the etiology of obesity and offer an opportunity to prevent or treat obesity by its therapeutic modulation. However, it is still a matter of debate to define which “indicator” microbial group is responsible for causing obesity as there are many contradictory findings with regard to the presence or absence of a particular microbiota in obesity. The discrepancies observed in the findings might be due to genetic background of host, age, sex, gut transit time, geographical location, and the diverse nature of gut microbiota. We believe that an in-depth study of gut microbiota at functional levels, i.e., metagenomics studies, along with focus on meta-transcriptomics and meta-proteomics, would provide an improved view of the picture by correlating the interlinked mechanisms. The outcomes will definitely help in understanding the known as well as unknown metabolic functions adhered by the gut microbiota of the host in leading to or preventing obesity.

### Gut Microbiota Link with Obesity: Mechanistic Insight

Gut microbiota play several crucial roles in host physiology such as immune modulation, digestion of indigestible food materials, and production of vitamins, bile acids, bioactive compounds [conjugated linoleic acid (CLA), bacteriocins]. They are also known to be involved in the degradation of toxins, carcinogens, inhibition of enteric pathogens, and maintenance of intestinal epithelia, all of which the host cannot achieve alone (Cani et al., 2013). It is proved that dysbiosis (imbalance in microbial community due to pathological state) of gut microbiota leads to the progression of several diseases in human beings such as obesity, diabetes, NAFLD), certain form of cancers, and even anxiety and depression (Luna and Foster, 2015; Leung et al., 2016; Perez-Chanona and Trinchieri, 2016). Therefore, understanding the relationship between host physiology and gut microbiota would pave new therapeutic opportunities. In the next section, we will describe the various mechanisms by which gut microbes influence host physiology, metabolism and energy storage, thereby making it susceptible to obesity. Yet, the interplay of these mechanisms and how they affect the overall metabolic status of an individual is not fully understood.

#### Gut Microbiota in Energy Harvesting from Indigestible Food

As our digestive system is deprived of enzymes to digest higher polysaccharides such as cellulose, xylan and pectin, upon ingestion, they reach the distal gut where these are fermented by the action of microbiota lying there. Actual digestion depends upon the type of microbial composition. *Bacteroides* are the dominating anaerobes there, which digest these polysaccharides, and in this context the starch hydrolytic system of *Bacteroides thetaiotaomicron* has been studied extensively. The simple sugars released after the fermentation of complex polysaccharides were influxed into glycolysis to generate ATP (adenosine triphosphate). Further hydrolysis of these biological molecules, which are produced by different microbial fermentation pathways, lead to the generation of more ATPs and simple carbon molecules. Of the SCFAs, acetate, propionate and butyrate are the most important end products of gut-situated microbial species (Koh et al., 2016) and absorbed in the body by passive diffusion and the action of mono-carboxylic acid transporters (MCT). Nearly 10% of the daily energy requirement by the host colonic epithelial cells and more than 70% of energy for cellular respiration is obtained from SCFAs. Among SCFAs, butyrate is the most liked source of energy for colonic epithelial cells (Kasubuchi et al., 2015). Persistent acquisition of energy from SCFAs leads to extra fat deposition in the body, which leads to obesity. However, the human diet varies greatly in fiber composition and that significantly alters the SCFA production. Studies of obese animal models showed an increased presence of SCFAs in the fecal material and similar findings was observed in human subjects. A reduced butyrate level was recorded in the fecal material of obese human subjects, who received varied carbohydrate content as part of their diet. Besides, a significant reduction in the population of *Roseburia/Eubacterium* rectal was also observed, which signified the important role of this group in butyrate formation (Louis and Flint, 2009). However, there lies a controversy over this matter as production of SCFAs from indigestible material depend on several factors in the gut environment such as availability of substrate, mucosal absorption, transit time of food, and interactions between different gut microbial species (Duncan et al., 2007). In addition to their role in providing energy, SCFAs also reduce the pH of the gut, thereby altering the composition of microbiota. An increase in pH from 5.5 to 6.5 reduces the abundance of butyrate producers and simultaneously increases the population of propionate producers. At a slightly acidic pH (at 5.5), proportions of *Firmicutes* was found to be predominated that is responsible for butyrate production. Whereas at pH 6.5, the population was predominated by *B. thetaiotaomicron*, which produced propionate as fermentation product (Duncan et al., 2009). These findings suggest that a particular microbial group outclasses another group/species for carbohydrates’ utilization at a specific luminal pH. However, these studies are confounding in nature and exact mechanisms are yet to be established.

#### Gut Microbiota Influence Fatty Acid Oxidation

Adenosine monophosphate kinase (AMPK), which is an important enzyme expressed mainly in the liver and skeletal muscles, plays a crucial role in cellular energy homeostasis. Drugs that increase the expression of AMPK lead to increase in fatty acid oxidation in liver and muscle tissues, incites energy loss, and disfavor obesity (Kim et al., 2017). Activation of AMPK eventually triggers carnitine palmitoyltransferase-1 (Cpt-1) *via* acyl-CoA carboxylase (Acc) activity, which in turn enhances mitochondrial fatty acid oxidation and inhibition of anabolic pathways such as glycogen storage and improved insulin sensitivity (Angin et al., 2016). Inhibition of AMPK by gut microbiota negatively influences fatty acid oxidation in target organs and tissues, promotes the synthesis of cholesterol and TG, and favor lipogenesis, which leads to excess fat storage and obesity (Boulangé et al., 2016). The fact was well understood by an experiment in which GF mice on a Western type diet had higher levels of phosphorylated AMPK, ACC and CPT-1 in the liver and skeletal muscles in comparison with conventionally raised mice. These elevated levels result in increased fatty acid oxidation in target tissues (Bäckhed et al., 2007). From here, it is inferred that gut microbiota have a suppressive effect on AMPK activity, which in turn affect fatty acid oxidation and make the host susceptible to obesity.

#### Gut Microbiota Influences Fasting Induced Adipose Factor (FIAF)

Fasting induced adipose factor, also called Angiopoietin-like 4 protein (ANGPTL4), is produced by adipose tissue, liver, skeletal muscle and intestine in response to fasting. It is also a powerful metabolism and a adiposity regulator (Dutton and Trayhurn, 2008). It is the main site of action for Peroxisome proliferator-activated receptor proteins (PPARs). Its main role is the inhibition of LPL, which in turn restricts TG accumulation in adipocytes (Wang and Eckel, 2009). Bäckhed et al. (2004) found that when GF mice were transplanted with the distal gut microbiota of conventionally grown mice, a 60% increase in the epididymal body fat was determined. They proposed that the transferred gut microbiota suppressed the FIAF expression in intestinal epithelium that in turn caused enhanced fatty acid uptake by adipocytes *via* increased LPL activity (Bäckhed et al., 2004). Further, the same group reported that GF *Fiaf*-/- mice were not protected from the development of obesity in comparison with their normal GF littermates fed on the same HFD. They concluded that the gut microbiota in wild-type GF mice suppressed the expression of *FIAF*, thereby increasing LPL activity and fat storage in adipocytes. In addition, the authors highlighted that Fiaf might modulate fatty acid oxidation in gastrocnemius muscle by means of controlling the expression of peroxisomal proliferator activated receptor co-activator 1α, which (Pgc1α) is accountable for coactivating every recognized nuclear receptors as well as many other transcription factors involved in mitochondrial fatty acid oxidation, including Cpt1 and medium-chain acyl-CoA dehydrogenase (Bäckhed et al., 2007). Thus, gut microbiota induces obesity with the help of the above-explained mechanisms. However, there lies a piece of evidence, which suggests gut microbiota are not able to provide resistance against obesity development or modulation in circulation of Fiaf/Angptl 4 levels. When GF mice and conventional mice were raised on HFD and Western type diet, then more weight gain was observed in GF mice on both the diets in comparison with their conventional littermates. The important thing was that this weight gain in GF was associated with increased intestinal mRNA levels of fasting-induced Fiaf/Angptl4, but not with circulating Fiaf/Angptl4. The population of gut microbiota was also found changed among conventional mice fed on HFD and wild-type diets. Thus, the study found that diet modulates the type of gut microbiota, and intestinal Fiaf/Angptl 4 does not have a crucial role in adipocytes’ fat storage as suggested by others (Fleissner et al., 2010). Therefore, the matter concerning the gut microbiota influence on *Fiaf* levels in obesity is still open for debate.

#### Gut Microbiota Influences Bile Acids

Bile acids are significant physiological molecules that facilitate digestion and absorption of fats in the small intestine and aid in the removal of lipids and toxic metabolites in the feces. Cholic acid (CA) and chenodeoxycholic acid (CDCA) are the main primary bile acids synthesized in the liver from cholesterol and are conjugated with taurine or glycine to form bile salts prior to secretion in bile. After their secretion into the intestinal lumen, these are converted into secondary bile acids deoxycholic acid and lithocholic acid by the dehydroxylation activity of bacteria. Subsequently, these bile acids are reabsorbed from ileum via ileal bile acid transporter (IBAT) through active transport and passive diffusion into the upper small intestine and colon. They are then transported back to the liver *via* blood circulation for re-secretion and feedback inhibition of bile acid synthesis in a process known as enterohepatic circulation. In this way, the bile acids affect intestinal absorption of fats, lipogenesis and ultimately metabolic homeostasis. Swann et al. (2011) demonstrated that mice having a distinct microbial structure in the gut possess different bile acid metabolites in their organs and hence have a divergent energy metabolism (Swann et al., 2011). Although, the underlying molecular mechanism of bile acid feedback inhibition is still not clear, but it has been suggested that nuclear receptor farnesoid X receptor (FXR) plays an important role in this regulation. FXR negatively regulates the expression of two key genes, namely, cholesterol 7a-hydroxylase (*CYP7A1*) and *CYP27A1*. *CYP7A1* is required for the initiation of classic pathways of bile synthesis while *CYP27A1* is required for the alternative pathway (Chiang, 2009). Recent studies have shown that intestinal FXR regulates hepatic CYP7A1 with the help of a fibroblast growth factor 15 (FGF15)-dependent mechanism (Zimmer et al., 2012). Sayin et al. (2013) in their re-derivation study of *FXR*-/- mice to GF showed that gut microbiota regulate expression of *FGF15* and *CYP7A1* by FXR-dependent mechanisms. The outcomes from this study suggest that the gut microbiota inhibits bile acid synthesis in the liver by alleviating the levels of FXR in the ileum (Sayin et al., 2013). Another mechanism by which bile acids regulate energy metabolism is by activating the G-protein-coupled bile acid receptor 1 (GPBAR1) or TGR5. This protein gets activated by interacting with secondary bile acids, as ligands, present in the intestinal lumen, thereby aiding in glucose homeostasis by activating secretion of glucagon-like peptide 1 (GLP1; Aron-Wisnewsky et al., 2013). Thus, in this manner, gut microbiota modulate bile acid metabolism by influencing FXR/TGR5 signaling and indirectly contributing toward the development of obesity. In addition, it is well known that bile acids exert an antimicrobial effect on gut microbiota by damaging the cell membrane integrity and thus its pool size and composition are considered as significant factors in the gut microbial community structure regulation. Composite and important alterations in the microbiome structure of animals were noticed when they were administered with bile acids (Ridlon et al., 2014). From these studies, it can be inferred that decrease in the levels of bile acids in the gut favors the population of gram-negative members, including some important pathogens. Conversely, an increase in bile acid amounts in the gut seem to promote gram-positive members of the *Firmicutes*, which include those bacteria that convert host primary bile acids to toxic secondary bile acids by 7α-dehydroxylation (Ridlon et al., 2014).

#### Gut Microbiota Influences Satiety

Apart from the role of SCFAs as substrate in energy metabolism, they also function as ligands for some receptors. Of those receptors, G-protein-coupled receptors; GPR41 (now called as FFAR3) and GPR43 (now called as FFAR2) are important target receptors. FFAR3 is expressed by the host immune cells, adipose tissue, spleen, bone marrow, large intestine, liver, and skeletal muscle (Le Poul et al., 2003; Regard et al., 2008). FFAR3 is mainly triggered by the presence of propionate, followed by butyrate and acetate, whereas FFAR2 is stimulated by all three SCFAs at the same rate (Brown et al., 2003). Notably, the presence of these receptors in different peripheral tissues clearly indicates that these SCFAs can directly influence several different functions such as satiety and host metabolism. One of the underlying mechanisms by which SCFAs regulate food intake, and satiety are *via* modulation of intestinal enteroendocrine L cells derived peptides, mainly GLP1 and peptide YY (PYY). These cells are found in abundance in the ileum and colon (De Silva and Bloom, 2012). The function of PYY is to reduce appetite by acting upon neuropeptide Y (NPY), thereby inhibiting gastric motility and reducing food intake (Karra et al., 2009). Likewise, the functions of GLP1, an incretin, are to regulate appetite, inhibit gastric emptying, and at the same time stimulate insulin secretion (Steinert et al., 2016). Nøhr et al. (2013) demonstrated that SCFAs activate GLP1 and PYY *via* stimulation of FFAR3 and FFAR2 present on L cells. These findings let us postulate that SCFAs produced from dietary polysaccharides, as a result of gut microbial fermentation, have direct influence on L cells, which in turn results in the rise of intestinal and plasma GLP 1 level. It is well documented in animal and human studies that ingestion of indigestible polysaccharides upregulates total GLP1 and PYY levels through SCFAs (Zhou et al., 2008; Tarini and Wolever, 2010). Tolhurst et al. (2012) reported that *FFAR2* or *FFAR3* knockout mice had reduced levels of GLP-1 and impaired glucose tolerance *in vitro* and *in vivo* at the same time due to lack of interaction with SCFA ligands. In a different gene knockout study, the authors revealed that mice lacking *FFAR2* gene became obese even after receiving a normal diet, while mice overexpressing *FFAR2* in adipose tissue stayed lean even after receiving a HFD. In addition, FFAR2 also suppresses insulin-mediated fat accumulation, which in turn regulates the energy balance by inhibiting the deposition of excess energy and inducing fat consumption (Kimura et al., 2013). Another mechanism, by which gut microbiota modulate energy homeostasis *via* SCFAs is their effect on leptin secretion from adipocytes through GPR41/43 dependent process. Thus, SCFAs and GPR41/43 interplay the role of significant messengers amidst gut microbiota and host metabolism (Xiong et al., 2004; Zaibi et al., 2010).

#### Gut Microbiota Influences Lipogenesis

The first experimental evidence that demonstrated that gut microbiota promote *de novo* hepatic lipogenesis came from the study of Bäckhed et al. (2004) on GF mice. In their pioneering research, the authors observed that transplantation of gut microbiota from normally raised mice to GF mice helps in inducing excess body fat storage and insulin resistance within the first 2 weeks despite reduced food intake. In subsequent years, another group studied the influence of gut microbiota on energy and lipid metabolism of host by comparing the serum metabolome and the lipidomes of serum, adipose tissue, and liver of conventionally raised and GF mice with the help of the MS-based metabolomics approach. Conventionally raised mice had an increased number of energy metabolites (e.g., pyruvic acid and citric acid) in their serum while, the levels of cholesterol and fatty acids were reduced. Moreover, they found that microbiota altered a number of lipid species in serum, adipose tissue, and liver, with the effect, mainly visible on TG and phosphatidylcholine species (Velagapudi et al., 2010). Enhanced TG synthesis observed was associated with an increase in the expression of the lipogenic genes, mainly acetyl-CoA carboxylase (Acc1) and fatty acid synthase (Fas). Both Acc1 and Fas are transcriptional sites of two key transcription factors, sterol response element binding protein 1c (SREBP-1c) and carbohydrate response element binding protein (ChREBP), required for lipogenesis in liver in response to insulin and glucose (Bäckhed et al., 2004). In conventionally raised mice, a significant enhancement in the levels of ChREBP was found in the liver as well as in the nucleus after its nuclear translocation, followed by its dephosphorylation by PP2A. Noticeably, PP2A was successively activated by xylulose-5-phosphate (Xu5P), an intermediate in the hexose monophosphate shunt. Conventionally raised mice reported to have higher levels of liver Xu5P compared with their GF littermates, suggesting that enhanced levels of this hexose monophosphate shunt intermediate further promote the liver ChREBP levels and consequently, liver lipogenesis.

These findings suggest that with an increase in fermentation of dietary polysaccharides, with the help of microbes, in conventionally raised mice, there is an increased supply of monosaccharides to the liver, which subsequently increases the activation of lipogenic enzymes by ChREBP and perhaps SREBP-1. The liver has two ways to tackle this increased influx of calories: it either increases the inefficient metabolism (futile cycles) or stores these surplus calories as fat in peripheral tissues (Bäckhed et al., 2004). Further, another research group demonstrated that gut microbiota induces *de novo* lipogenesis and TG synthesis in HepG2 cells by production of t10,c12 CLA. They found that treating cells with t10,c12 CLA increased lipid deposition *via* increased incorporation of acetate, palmitate, oleate, and 2-deoxyglucose into TG. CLA treatment also led to upregulate the mRNA expression as well as protein levels of lipogenic genes, including *SREBP1*, *ACC1*, *FASN*, *ELOVL6*, *GPAT1*, and *DGAT1*, thereby presenting a potential mechanism by which CLA increased lipid accumulation. Most importantly, CLA treatment also increased the phosphorylation of mTOR, S6K, and S6. Together, the authors concluded that t10,c12 CLA production by gut microbiota induces liver *de novo* lipogenesis and TG synthesis is linked with the activation of the mTOR/SREBP1 pathway that consequently, leads to increased lipid incorporation in HepG2 cells (Go et al., 2013).

#### Gut Microbiota and Innate Immunity

Toll-like receptors (TLRs) are groups of proteins that play an important role in the innate immune system. They are membrane-spanning, non-catalytic receptors normally expressed on sentinel cells that recognize structurally conserved motifs of microbes called pathogen-associated molecular patterns (PAMPS) (Medzhitov, 2001). The interaction of these PAMPS with host TLRs induces several antimicrobial immune responses through the activation of inflammatory signaling pathways that are necessary for the effective immune response. Therefore, there is no doubt about the fact that the microbiota we harbor in our gut, and which interacts with epithelium TLRs at the luminal interface, is vital for maintaining the immune homeostasis (Peterson et al., 2015). Of the various PAMPS of bacteria, TLR5 mainly detects bacterial flagellin from invading bacteria and are found highly expressed in the intestinal mucosa. Vijay-Kumar et al. (2010) elucidated the role of TLR5 receptor in adiposity progression and associated metabolic syndrome. They found that TLR5 deficient mice (TLR5KO) exhibited many features of metabolic syndrome such as hyperphagia, hyperlipidemia, hypertension, hypercholesterolemia, high blood pressure, insulin resistance, and enhanced fat deposition in comparison with normal counterparts. They demonstrated that these changes were associated with an increase in adipocytes secretion of proinflammatory cytokines IL-1β and INF-γ (Vijay-Kumar et al., 2010). Next, the authors examined whether changes in the gut microbiota, resulting from loss of TLR5, helped in the development of metabolic syndrome. In order to do so, they placed TLR5KO mice and wild-type littermates on antibiotics and found that destruction of gut microbiota in TLR5KO mice ameliorated metabolic syndrome similar to wild-type mice. UniFrac analysis showed that the gut microbiota composition of TLR5KO, and wild-type littermate mice was remarkably different. Besides marked inter-individual differences in species diversity, they observed 116 bacterial phylotypes from various phyla to be consistently enriched or reduced in TLR5KO mice in comparison to wild-type mice (Vijay-Kumar et al., 2010). To further assess whether alteration in the gut microbiota was a factor responsible for the development of metabolic syndrome in TLR5KO mice, they transplanted the microbiota from TLR5KO mice to wild-type, GF mice. They found that the transplanted microbiota conferred many phenotypic effects of TLR5KO to wild-type mice. The authors concluded that the gut microbiota play a crucial role in the development of metabolic diseases and opined that dysfunction of the innate immune system may be one factor that favor their development. However, there is one study in which TLR5KO mice from two different animal colonies, neither exhibited evidence of metabolic abnormalities nor showed enhanced basal intestinal inflammation (Letran et al., 2011). Therefore, the authors concluded that basal inflammatory phenotype is not a consistent feature of TLR5-deficient mice.

#### Gut Microbiota, Metabolic Endotoxinemia and the Endocannabinoid System

The progression of obesity is associated with the activation of low grade inflammatory signaling molecules from adipose tissue such as TNF-α, IL-1, IL-6, which disrupt normal metabolic processes and mediate insulin resistance (Hotamisligil, 2006; Ouchi et al., 2011). The adverse effects of insulin resistance lead to hyperinsulinemia, and excessive hepatic and adipose tissue storage of fat. For a long time, however, the inflammation triggering molecules in HFD-induced obesity remained unknown and it was Cani et al. (2007a) who first proposed that a Gram-negative bacterial outer membrane component known as lipopolysaccharide (LPS) was responsible for early onset of inflammation, insulin resistance, obesity and diabetes (Cani et al., 2007a). The authors found that supplementation of HFD in mice for 4 weeks chronically increased plasma LPS levels 2- to 3-fold than those of control animals and called it “metabolic endotoxemia.” Notably, increased LPS levels in the HFD group were associated with a decreased abundance of *Bacteroides*, *Eubacterium rectale*-*Clostridium coccoides* group and *Bifidobacterium* species. In a subsequent set of experiments, the authors subcutaneously infused LPS in GF mice for 4 weeks and found that changes in body weight, metabolic physiology, and endotoxemia were similar to the ones earlier seen with HFD. However, the effect of LPS-induced metabolic changes was diminished when the mice were made deficient in the genes *cd14* and *tlr4* (Cani et al., 2007a; Davis et al., 2008; Vijay-Kumar et al., 2010). This signifies that LPS induces systemic inflammation *via* these markers. Next, to assess whether modulating the gut microbiota could control the occurrence of metabolic endotoxemia and the resultant metabolic diseases, the authors made use of antibiotics on intestinal microbiota of HFD and genetically obese (*ob*/*ob*) mice. As a result, a decrease in inflammation, obesity-related bio-markers and endotoxemia levels were observed. Noticeably, high fat feeding also increased intestinal permeability and simultaneously reduced the expression of genes coding for two tight junction proteins ZO-1 and occludin (Cani et al., 2008). HFD dramatically decreased the population of *Lactobacillus* spp., *Bifidobacterium* spp., and *Bacteroides–Prevotella* spp. Interestingly, feeding of *bifidobacteria* reversed metabolic endotoxemia, and improved gut integrity and associated metabolic changes in mice (Wang et al., 2006; Cani et al., 2007c). However, no relationship was found between endotoxemia and other bacterial groups *E. rectale*–*C. coccoides, lactobacilli–enterococci, Bacteroides*, and sulfate-reducing bacteria (Cani et al., 2007c). Until this point, no information was available concerning molecular mechanism that linked how modulation in gut microbiota improved metabolic endotoxemia, tight junction integrity, obesity-related hepatic and metabolic disorders. Therefore, to decipher the underlying mechanism, Cani et al. (2009b) performed three different sets of experiments on genetically obese mice (*ob/ob*) using different strategies. In the end, they found that selective modulation of gut microbiota by probiotic supplementation regulates and enhances the endogenous production of intestinotrophic GLP-2, which in turn improves gut barrier integrity and functions by way of a GLP-2-dependent mechanism during obesity and diabetes (Cani et al., 2009b). In addition, they advocated the role of the endocannabinoid (eCB) system in gut barrier integrity and obesity. The eCB system consists of eCBs, their receptors, and enzymes that synthesize and degrade eCBs (Mackie, 2008). Cannabinoid receptor type 1 (CB1) and type 2 (CB2) are two important G-protein-coupled cannabinoid receptors activated by the eCB system. Two eCBs, namely anandamide and 2-Arachidonoylglycerol (2-AG), play a significant role in adipogenesis by activating their receptors. Anandamide activates CB1 while 2-AG activates both cannabinoid receptors. The eCB system was found hyperactive (greater system tone) in case of obesity and type 2 diabetes. It has been seen in several studies that there is a close connection between LPS and the eCB system. In fact, some *in vitro* and *in vivo* studies reflect that LPS regulates the synthesis of eCBs *via* LPS receptor-mediated signaling pathways (Muccioli et al., 2010). But the influence of gut microbiota on eCB signaling was yet to be understood. Muccioli et al. (2010) found that gut microbiota modulate the intestinal eCB system tone, which regulates gut permeability and plasma LPS levels. Besides, they also showed that LPS plays a central role in adipose tissue metabolism both under *in vivo* and *in vitro* by blocking cannabinoid-driven adipogenesis. From their study, it could be figured that gut microbiota regulate adipogenesis through LPS–eCB system loop (Muccioli et al., 2010).

In subsequent studies, the same research group tried to investigate the effect of eCB, LPS, and the gut microbiota in the regulation of apelin and APJ expression in adipose tissue (Geurts et al., 2011). Apelin and APJ are found widely expressed in mammalian tissues and deploy their functional effects both in the central nervous system and in the peripheral nervous system. Apelin is the endogenous ligand for the G-protein-coupled receptor known as APJ receptor. Apelin was found to play a significant role in the cardiovascular system by acting on heart contractility, blood pressure, fluid homeostasis, vessel formation, and cell proliferation (Maenhaut and Van de Voorde, 2011). Interestingly, apelin also acted on glucose homeostasis via AMP-kinase- and nitric oxide (NO)-dependent mechanisms (Dray et al., 2008). At the end of the study, the investigators came up with the inferences that apelin and APJ expressions were suppressed by the eCB system in physiological conditions and increased by LPS in pathological situations such as obesity and diabetes (Geurts et al., 2011).

**FIGURE 1:**
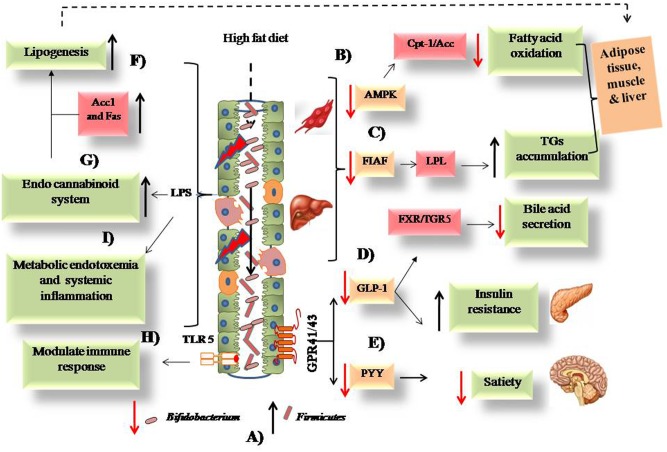
**Possible mechanisms associated with the intake of high fat diet and obesity. (A)** High fat diet causes alteration in intestinal microbiota from low to high *Firmicutes* and high to low *Bifidobacterium*. **(B)** Low expression of AMPK leads to decreased fatty acid oxidation. **(C)** FIAF expression causes activation of LPL that leads to TGs accumulation. **(D)** Low GLP-1 leads to increased insulin resistance and decreased bile acid secretion from liver. **(E)** Decreased PYY causes low satiety in obese host. **(F)** Increased lipogenesis via upregulated Acc1 and Fas enzymes. **(G)** Activation of endo cannabinoid loop via release of LPS due to damages intestinal epithelium. **(H)** Modulation of intestinal immune response via TLR-5 downstream signaling. **(I)** Systemic inflammation caused by inflammatory cytokines and bacterial LPS.

Thus, it seems that several factors play important roles in the regulation of gut permeability and adiposity, among which the role of LPS is visualized as central to all these mechanisms. All the above proposed mechanisms are represented in a pictorial manner in **Figure 1**.

## Modulation of Gut Microbiota by Dietary Approaches for Therapeutic Effects

From the aforementioned studies, it can be inferred that gut microbiota plays a central role in host physiology in obesity. Therefore, it is quite feasible to hypothesize that its positive modulation by external approaches may provide beneficial effects to the host. Out of the available intervention approaches (diet, antibiotics, surgery), dietary strategy is much preferred by medical practitioners due to associated lesser cost and safety issues. Future therapeutic strategies can be formulated by understanding which dietary substance has a positive modulatory effect. Probiotics and prebiotics are promising because of their direct influence on the gut microbiota. In the coming sections, we have described the effect of prebiotics and probiotics on the gut microbiota and their outcomes in experimental settings (animal and human). However, in the past few decades, fecal transplantation of the gut microbiota has also gained momentum, but this practice is only limited to some countries or to certain research laboratories/institutions and are not discussed here in this review.

### Prebiotics in Modulation of Gut Microbiota in Context to Obesity

#### Evidence from Animal Studies

Prebiotics are the indigestible dietary polysaccharides that promote the growth of inherited gut microbes or probiotics when supplied externally. The most commonly used prebiotics in practice are fructooligosaccharides, galactooligosaccharides, lactulose, and non-digestible carbohydrates inulin, cellulose, resistant starches, hemicelluloses, gums, and pectins because they fulfill the criterion as suggested by Gibson et al. (2004). The science of using prebiotics for therapeutic applications is not new as they were used by our elders to assist in restoring people back to health when diseases struck. But the science picked up pace during the past few decades, when Cani et al. (2004) found that inulin type dietary fructans (ITF) [oligofructose (OFS) and Synergy 1] have the potential to increase intestinal proglucagon and GLP-1 levels, and simultaneously decrease the expression of ghrelin in the treated Wistar rat than the control. These gut hormones are critically involved in the regulation of appetite and body weight in human and animal models (Cani et al., 2004). A similar hypothesis was tested among HFD fed Wistar rats administered with OFS as prebiotic. Consequently, feeding of OFS provides obesity ameliorating effect in rat due to modulation in the expression of gut situated peptides as described in a previous finding except for GLP-2. However, the exact mechanism how these prebiotic fibers made changes in secretion of orexigenic- and anorexigenic peptides, and thereby changes in the energy homeostasis was not elusive, but proposed due to activity of SCFAs that promoted the production of these peptides from endocrine L cells (Cani et al., 2004, 2007b). Later on, the concept incepted that these prebiotic fibers modulate the microbial community upon ingestion in gut in particular of *bifidobacteria* and *lactobacilli* (Everard et al., 2011; Neyrinck et al., 2011; Gérard, 2016). In a meta-analysis, review concerning the modulation of gut microbiota by prebiotics and probiotics to counter obesity, the authors found that in most of the studies, *bifidobacteria* plays a central role in ameliorating obesity by promoting its growth in presence of prebiotics (da Silva et al., 2013). However, there is a study which has shown that the stimulating effect of prebiotics is not only restricted to *bifidobacteria*, *lactobacilli*, and *F. prausnitzii*, but also influences other bacterial taxa that play an important role in obesity (Respondek et al., 2013; Everard et al., 2014). Notably, more often, this alteration in gut microbiota by prebiotic induction provides obesity reducing effects by indirectly acting on various pathological sites responsible for the development of obesity.

Cani et al. (2006b, 2009b) found that feeding OFS to HFD mice led to a considerable increase in the bifidobacterial count, which in turn decreased the inflammatory markers by way of reduced LPS production. Decreased LPS production improves gut permeability and reduces adiposity. Later on it was elucidated that low metabolic endotoxemia resulted because of the bifidogenic effect of prebiotic. As these fibers increase the expression of gut hormones GLP-1 and GLP-2 from L cells and also modulate the eCB system; these modulations in-turn alleviate inflammation and insulin resistance in mice (Cani et al., 2006b, 2009b; Muccioli et al., 2010). In addition to *Bifidobacteriaceae*, the impact of prebiotic feeding on other gut microbiota was also revealed with the help of molecular biology approaches. Prebiotic feeding in genetically obese mice led to a decrease in *Firmicutes*, while registering an increase in *Bacteroidetes*. Change in proportion of more than 100 distinct taxa was also revealed, out of which 16 taxa displayed more than a 10-fold change. This led to the identification of *A. muciniphila*, whose population in the gut is negatively correlated with obesity (Everard et al., 2011). They hypothesized that *A*. *muciniphila* has a positive role in obesity, that was validated by a recent finding wherein feeding of bacteria to dietary HFD mice provided alleviation of pathophysiological parameters and reduction in body weight (Schneeberger et al., 2015). In addition to modulation of gut microbiota, prebiotic feeding also increases the number of L cells and positively modulates the various parameters (GLP-1, fat mass development, oxidative stress, etc.) responsible for the development of metabolic syndromes. The researchers unraveled a new mechanism linking gut microbiota-mediated change in metabolism of genetically obese mice in which feeding of prebiotics had improved leptin sensitivity (Everard et al., 2011). Subsequently, the mechanism by which *A*. *muciniphila* plays an important role in the amelioration of obesity, and related disorders was elucidated by Everard et al. (2013). Prebiotic feeding stimulates the growth of *A. muciniphila* that concomitantly increases the intestinal levels of eCB, which regulates inflammation, gut permeability, and anorexigenic peptide. However, only viable cells of *A. muciniphila* can address these effects.

#### Evidence from Human Studies

If we talk about the impact of prebiotic (inulin type) supplementation on healthy human physiology, then they have been reported to induce satiety, increase breath-hydrogen excretion, modulate gut peptides involved in appetite regulation (Cani et al., 2006a, 2009a; Parnell and Reimer, 2009), and prompted the growth of *bifidobacteria* and *lactobacilli* (Gibson et al., 2004). Whether these prebiotic (inulin) stimulated the growth of whole *bifidobacteria* genus or a particular species or other members of human gut microbiota was unknown. Ramirez-Farias et al. (2009) found that inulin ingestion specifically stimulated the growth of *B. adolescentis* among other analyzed species. Besides, *F. prausnitzii* was found as bacterial species other than lactic acid bacteria that was stimulated because of inulin ingestion. However, the study is not elusive because of the involvement of only a few volunteers in the study. In a similar finding, Joossens et al. (2011) reported that ingestion of OFS-enriched inulin to 17 human volunteers led to the significant increase in *B. longum* and *B. adolescentis* species. A later prebiotic intervention study in obese women provided an insight of the effect of this treatment on the gut microbiota. Inulin type prebiotics promoted growth of *Firmicutes* and *Actinobacteria*, and inhibition of *Bacteroidetes*. A deeper analysis revealed that there was an increase in the population of *Bifidobacterium* and *F. prausnitzii*, while a decrease was noticed in *Bacteroides intestinalis* and *B. vulgatus*, after prebiotic treatment. Despite that increase in the population of *lactobacilli* was also observed after prebiotic treatment. From the correlation analysis between prebiotic treatment and host metabolism, it could be speculated that *Bifidobacterium* and *F. prausnitzii* were negatively correlated with serum LPS levels, while changes in *B. intestinalis* and *B. vulgatus* and *Propionibacterium* were positively correlated with changes in body composition and glucose homeostasis (Dewulf et al., 2012). In conclusion, the authors suggested that treatment with ITF prebiotics alleviated host obesity related mechanism *via* selective modulation in the gut microbial signatories of obese women. In a subsequent study, the investigator tries to establish a correlation between *Bifidobacterium* species, SCFAs, and key metabolic markers of host physiology. Ingestion of ITF by obese women led to an increase in the population of *B. longum*, *B. pseudocatenulatum*, and *B. adolescentis*. Modulation in numbers of *B. bifidum* and *B. adolescentis* was inversely linked with fat mass percentage, while *B. breve* was negatively correlated with serum cholesterols. Strikingly, *B. longum* was negatively linked to serum LPS. The levels of SCFAs (acetate and propionate) were also found to be low in treatment groups compared with control ones. In summary, the authors affirmed that ingestion of ITF prebiotics in obese women led to an increase in the population of *Bifidobacterium* species and a decrease in the production of SCFAs, which ultimately reduce the host metabolic parameters associated with obesity (Salazar et al., 2015).

However, it is predicted that instead of SCFA other metabolites (bile acids, choline, vitamins, polyamines, and lipids) produced by gut microbiota under influence of prebiotics also have a significant role in the host physiology. It is reflected from a finding wherein authors fed a HFD and prebiotic rich diet (ITF or arabinoxylans) to mice and found an increase in the rumenic acid (*cis*-9, *trans*-11-18 : 2 CLA) content in both the caecal and liver tissues compared with the control group. Of the two prebiotics tested, only arabinoxylans were able to increase the rumenic acid content because their prebiotic fibers might have provided high fat-binding capacity which provides more substrates for bacterial metabolism to differentially modulate the gut microbiota. Rumenic acid is produced from linoleic acid by gut microbes by their biohydrogenation activity during a detoxifying mechanism. A similar effect was also observed with gut isolated microbes when they were subjected to substrate linoleic acid during *in vitro* studies. In conclusion, the authors suggested that the CLA-producing bacteria could be a responsible for addressing the metabolic effects in both HFD feeding and prebiotic supplementation (Druart et al., 2013).

Altogether, prebiotics manage obesity by lowering the production of LPS by modulating the gut microbiota that ultimately hinders the process of low grade inflammation and modulates the eCB system. They also reported to induce satiety via promotion of satiety peptides from L cells in the gut.

### Probiotics in Modulation of Gut Microbiota in Context to Obesity

#### Evidence from Animal Studies

Apart from prebiotics, there lies another alternative dietary approach in which probiotics are used to modulate gut microbiota. This method led to a rise in anti-obesity effects across animal and human studies. Probiotics are the live microorganisms which, when fed in adequate amount, confer health promoting effects on the host (Sanders, 2008). Members of lactic acid bacteria, namely *Lactobacillus* spp. and *Bifidobacterium* spp. are the two extensively studied probiotics that have provided anti-obesity effects in animal models and human beings (**Tables 1, 2**). However, these days only those strains that pass the prescribed probiotic and functional tests are used for animal and human use (Dahiya and Puniya, 2015). The proposed mechanism of action includes alteration in the gut microbial community, production of bioactive compounds by probiotic strains, reduction in fat storage, alterations in serum lipid profiles, induction in fatty acid oxidation genes, interaction of probiotics with host TLRs, reduced expression of pro-inflammatory cytokines, and stimulating the production of satiety-inducing peptides (Stanton et al., 2005; Tsai et al., 2014; Villena and Kitazawa, 2014; Dahiya and Puniya, 2017).

**Table 1:** Effects of probiotics on gut microbiota of animals and their physiological outcomes.

Probiotics	Animal model	Influence on gut microbiota	Metabolic outcome	Reference
VSL#3	C57J/B67 HFD and *ob/ob* mice	↓*Firmicutes*, ↑*Bacteroidetes*, and *bifidobacteria*. ^∗^ NC on *lactobacilli*	↓ Body weight (BW), food intake and adiposity, ↑ insulin sensitivity, glucose tolerance, production of GLP-1 and SCFA butyrate	Yadav et al., 2013
*L. curvatus* HY7601 and *L. Plantarum* KY1034	C57BL/6J HFD mice	NC in *Firmicutes* and *Bacteroidetes*. Phylum *Verrucomicrobia* was absent and ↓*Proteobacteria*. ↑ Four Species belonging to the *Ruminococcaceae* and *Lachnospiraceae* families of the order *Clostridiales* and phylum *Firmicutes*. ↑ endogenous *B. pseudolongum* was found higher	↓ BW and fat accumulation, plasma insulin, leptin, total cholesterol (TC) and liver toxicity biomarkers and adipose tissue, ↓ pro-inflammatory genes in adipose tissue while ↑ fatty acid oxidation genes in liver	Park et al., 2013
*L. salivarius* UCC118	C57BL/J6 HFD mice	NC in *Firmicutes*, ↑ *Bacteroidetes* and *Proteobacteria* and ↓*Actinobacteria*	No alteration in metabolic profiles	Murphy et al., 2013
*L. salivarius* UCC118	C57BL/J6 HFD mice	↑ In proportions of *Bacteroidetes* and *Peptococcaceae* and significantly ↓ in population of *Rikenellaceae* and *Porphyromonadaceae*	↓ BW at appropriate time	Clarke et al., 2013
*L. coryniformis* CECT5711	C57BL/6J HFD mice	↑ *Lactobacillus* spp. cluster	↓ Plasma glycaemia, insulin resistance and LPS levels and improves gut permeability.	Toral et al., 2014
*L. casei* NCDC 19	C57BL/6J HFD mice	NC in total bacterial counts, *Eubacterium rectale–Clostridium coccoides* and *lactobacilli–Enterococcus* (LAB) groups. Significant ↑ in population of *bifidobacteria* was observed	↓ BW, epididymal fat, blood glucose, plasma lipids and leptin; ↑ adiponectin	Rather et al., 2014
*L. brevis* OK56	C57BL/6J HFD mice	↑ *Bifidobacteria* population	↓ Body and epididymal fat, ↓ NF-κB activation and LPS production. ↓ Pro-inflammatory cytokines	Kim et al., 2015
*L. rhamnosus* NCDC17	High fat diet fed and streptozotocin treated Wistar rats	↑ *Bifidobacteria* and *lactobacilli* proportions	↑ Glucose tolerance, blood glucose, plasma insulin, glycosylated hemoglobin, FFA, TGs, serum lipids and cholesterol, oxidative stress. ↑ GLP1 and adiponectin, ↓ pro-inflammatory cytokines and propionate in caecum	Singh et al., 2016
Probiotic mixture (*L. salivarius* 33, *L. rhamnosus* LMG S-28148, *B. animalis* subsp. *lactis* LMG P-28149)	C57BL/6J HFD mice	↑ *Bacteroidetes*, Restores the adundance of *Rikenellaceae* ↓*Lactobacillaceae*. Probiotic treatment induced a modification in the *bifidobacterial* population: *B. pseudolongum* was no longer detected that was present in HFD group. ↓*Lactobacillus–Leuconostoc–Pediococcus* group. Restoration of *A. muciniphila* after treatment	↓ BW and adiposity with improvement in insulin resistance, ↓ adipose tissue inflammation, ↑ dyslipidemia through adipose tissue immune cell-remodeling	Alard et al., 2016
*L. plantarum* HAC01	C57BL/6J HFD mice	↑ *Lachnospiraceae*, ↓*Deferribacteraceae*, *Mucispirillum* and *Lactobacillaceae* with NC in proportion of *Firmicutes* and *Bacteroidetes*	↓ BW gain and mesenteric fat weight, blood glucose, TC and triacylglycerol, ↑ lipid oxidative genes	Park et al., 2017

*^∗^NC, No significant change.*

In most of the accomplished *in vivo* studies, gut microbiota was not studied, although modulation of gut microbiota by probiotic feeding presented an interesting therapeutic approach. Yadav et al. (2013) demonstrated that feeding of probiotic VSL#3 consortiums attenuate obesity and diabetes in mouse models *via* modulation of the gut flora. Deeper investigation revealed that VSL#3 stimulated the production of GLP-1 *via* butyrate production from altered gut microbiota, which addressed reduced food intake, improved glucose tolerance, and reduced adiposity. In another study, oral feeding of *L. curvatus* HY7601 and *L. plantarum* KY1032 to HFD mice significantly shifted the microbial communities, which ultimately reduced obesity in mice. The comparative abundance of four species belonging to the *Ruminococcaceae* and *Lachnospiraceae* families of the order *Clostridiales* and phylum *Firmicutes* decreased in the high fat control group and increased among the probiotics-administered mice. This microbial shift was accompanied with anti-obesity effects in mice that were probably due to induced positive influence on the expression of inflammatory and lipid oxidation markers situated in the liver and adipose tissue.

Murphy et al. (2013) demonstrated that feeding bacteriocin producing probiotic *L. salivarius* UCC118Bac+ to mice had the potential to alter their gut microbiota. The feeding of this strain to mice results in a relative increase in *Bacteroidetes* and *Proteobacteria*, decrease in *Actinobacteria*, but no effect on *Firmicutes* in comparison with non-bacteriocin producing strain. However, this strain was unable to address any change in the metabolic physiology of mice. In their subsequent investigation, the same group showed interest in elucidating the time dependent effect of feeding the *L. salivarius* UCC118Bac+ and a shift in the gut microbial composition. Initial treatment resulted in a significant increase in amount of *Peptococcaceae* and decrease amount of *Rikenellaceae* and *Porphyromonadaceae* in comparison with the gut microbiota of control mice. The findings highlighted the ability of gut microbiota to recover its shape after a period of time and require long term probiotic treatment to undergo sustained modification (Clarke et al., 2013).

Toral et al. (2014) showed that administration of *L. coryniformis* CECT5711 reduces gut dysbiosis that improves metabolic endotoxemia by lowering LPS levels and improving gut permeability, which thereby improves obesity in mice. Another study found that feeding of probiotic dahi, which contains *L. casei* NCDC 19, led to a reduction in epididymal fat weights, blood glucose, plasma lipids, leptin levels, and body weight among HFD mice (Rather et al., 2014). These observed effects were correlated with an increase in the population of *bifidobacteria*. Kim and co-workers found that administrating *L. brevis* OK56 to HFD mice abrogated the adverse effect of diet on gut microbiota. Despite the increase in population of *bifidobacteria*, OK56 supplementation suppressed colonic and plasmatic LPS and decreased production of H_2_ breath gas. The authors suggested that the anti-obesity effect exerted by OK56 was due to inhibition of LPS production by modulation of gut microbiota and suppression of other inflammatory pathways (Kim et al., 2015). Similar results were observed by Lim et al. (2016) who found that feeding *L. sakei* OK67 to HFD mice helped in ameliorating obesity by reducing production of LPS, which was possibly due to modulation of gut microbiota. They also opined that probiotic feeding induces the expression of tight junction proteins, which are responsible for maintaining gut integrity. In a recent finding, the authors found that feeding diabetic rats *L. rhamnosus* NCDC17 increases the population of *bifidobacteria* and *lactobacilli* in the cecum, although it also resulted in attenuation of other biomarkers responsible for development of obesity (Singh et al., 2016). Similar findings were also conducted by others. Alard et al. (2016) showed that adiposity dampens the effect of probiotics, which are linked to the improvement of dysbiotic gut microbiota. They observed that feeding a probiotic strain restores the abundance of *A. muciniphila* and *Rikenellaceae* and decreases *Lactobacillaceae*. These gut-associated alterations are linked with improvement in other pathological parameters and obesity (Alard et al., 2016). Recently, Park et al. (2017) demonstrated that feeding of a probiotic strain *L. plantarum* HAC01 to HFD mice resulted in reduction of body weight, fat mesenteric fat, and other biomarkers associated with obesity. In spite of these changes, significant alterations in several bacterial taxa, both on family and genus level, were observed, as revealed in metagenomics’ studies. HAC01 feeding led to a significantly increase in the abundance of the family *Lachnospiraceae* (phylum *Firmicutes*), while decreasing the population of *Deferribacteraceae.* The decrease in abundance was due to a significant reduction in genus *Mucispirillum* numbers. Interestingly, administration of HAC01 also resulted in a decrease in the population of *Lactobacillaceae.* Moreover, no remarkable change in the relative proportion of *Firmicutes* and *Bacteroidetes* was observed post treatment. Finally, the authors suggested that administration of probiotic strain induces modulations in the gut microbiota, which in turn influences the regulation of genes associated with lipid metabolism. These series of changes may consequently, abrogate the fat storage and alleviation of host metabolism (Park et al., 2017).

The above studies made us understand that probiotics address obesity, at least in animals, *via* modulation of gut microbiota. But clear-cut studies are still missing. Also, in most of the studies, only few bacteria tax or phylum or family or genus were studied and that too with the help of conventional techniques. Only one studied provided deeper inside of modulated gut microbiota under influence of probiotic treatment. The discrepancy between the studies may be due to differently inherited gut microbiota of the host of varied genetic background, age and diet. So, here we emphasized on the application of advanced “omics”-based techniques to study the changes in the gut microbiota in probiotic intervention studies, whereby particular “indicator” taxa instead of phyla can be linked with anti-obesity potential. Besides, mechanistic studies are also warranted that decipher how particular “indicator” taxa crosstalk with probiotic strains during reversal of obesity. Moreover, the effects of probiotics are strain specific, so exploring the impact of a single strain on gut microbiota modulation further improves our understanding in the context of host metabolism.

#### Evidence from Human Clinical Trials

Most of the human studies concerning the impact of probiotics on body weight were restricted to the analysis of biochemical (inflammatory markers) and physical parameters related to metabolic disorders (reviewed by Sanz et al., 2013). Only few studies have evaluated their effect on gut microbiota in the context of obesity and associated disorders (**Table 2**). Luoto et al. (2010) evaluated the impact of perinatal probiotic (*L. Rhamnosus* GG, ATCC 53103) feeding on childhood growth and development patterns up to a period of 10 years in follow-up study. The results signified that probiotic feeding during the early years modulated the gut microbiota of children, which in turn changed growth patterns by way of restraining excessive weight gain (Luoto et al., 2010). In a subsequent clinical trial, the authors studied the impact of *L. salivarius* Ls-33 supplementation on the fecal microbiota of obese adolescents. The administration significantly increased the ratio of *Bacteroides*–*Prevotella*–*Porphyromonas* group to *Firmicutes* belonging bacteria. The population of *Lactobacillus* spp. and *Bifidobacterium* spp. changed remarkably post feeding. Also, no change in the production of SCFAs was observed between treatment and placebo group. The authors concluded that the probiotic modulated fecal microbiota by a method not related to metabolic syndrome (Larsen et al., 2013). A later study was designed to assess the combined effect of probiotic capsules (*L. plantarum*, *L. acidophilus*, *L. rhamnosus*, *B. lactis*, *B. longum*, *B. breve*, and *Streptococcus thermophilus*) and herbal medicine in the treatment of obesity among patients having BMI > 25 kg/m^2^ and waist circumference >85 cm. The results demonstrated a major reduction in body weight and waist circumference, but no remarkable differences in body composition and metabolic biomarkers were noticed. When they correlated the change in body composition with LPS level and the population of gut *L. plantarum*, a positive relation was revealed. A positive correlation was also documented for Gram-negative bacteria with alterations in body composition and total cholesterol level. A negative correlation was found between *B. breve* population and LPS level. The conclusion corroborated the fact that probiotics play a significant role in deterring obesity by a reduction in LPS production through altered gut microbiota (Lee et al., 2014). In a subsequent study, the individual or symbiotic effect of probiotic *L. salivarius* UBLS22 and prebiotic (fructo-oligosaccharide) supplementation on the various biomarkers of obesity and gut microbiota in healthy young volunteers was examined. After treatment, significant positive alterations in the serum lipid profile were observed in the probiotic as well as symbiotic groups. The serum concentrations of inflammatory cytokines were also reduced in the two treatment groups. They observed a noteworthy boost in the population of *lactobacilli*, and a decrease in total coliforms and *Escherichia coli* across both groups. However, a more pronounced effect was observed in the symbiotic group than the individual one. The authors advocated that the symbiotic mixture could be used for the treatment of obesity by modulating the serum lipid profiles, inflammatory cytokines, and gut microbiota (Rajkumar et al., 2015).

**Table 2:** Effect of probiotics supplementation on human gut microbiota and their metabolic outcomes.

Probiotic and subject	Study Design	Influence on gut microbiota	outcome	Reference
*L. rhamnosus* GG, ATCC 53103 pregnant women and children	Double-blind placebo controlled Mothers: 4 weeks before deliver Children: 6 months after birth	No clear cut study on gut/fecal microbiota	The authors proposed that the reduction in BW was due to positive modulation of gut microbiota by probiotic during the critical development period.	Luoto et al., 2010
*L. salivarius* Ls-33 obese adolescents	Double-blind placebo controlled 12 weeks	aaa *Bacteroides–Prevotella*–*Porphyromonas* group to *Firmicutes* belonging bacteria, including *Clostridium* cluster XIV, *Blautia coccoides_Eubacteria rectale* and *Roseburia intestinalis* group. NC of ingestion on *Clostridium* cluster I/IV, *F. prausnitzii*, *Enterobacteriaceae*, *Enterococcus*, *Lactobacillus*, and *Bifidobacterium* group	Feeding strain might have modified the fecal microbiota in obese adolescents by a mechanism that is not associated to metabolic syndrome.	Larsen et al., 2013
Probiotic (*S. thermophiles*, *L. plantarum*, *L. acidophilus*, *L. rhamnosus*, *B. lactis*, *B. longum* and *B. breve)* and *Bofutsushosan* herb, subjects having BMI > 25 kg/m^2^ and waist circumference > 85 cm were only included in the study	Double-blind, placebo controlled 8 weeks	aaa *B. breve*, *B. lactis*, *L. rhamnosus*, and *L. plantarum*. ↓ *Firmicutes/Bacteroidetes* ratio was also observed upon treatment.	↓ Weight, waist circumference and aaa in HDL-cholesterol. Change in body composition is positively related to levels of LPS and *L. plantarum*	Lee et al., 2014
*L. salivarius* UBL S22 and Prebiotic Fructoligosaccharide healthy human volunteers	Double-blind, placebo controlled 6 weeks	aaa *lactobacilli* and ↓ *E. coli* after intervention period.	Improvement in serum TGs and lipid profile with ↓ in inflammatory cytokines	Rajkumar et al., 2015

From the aforementioned clinical trials, it can be inferred that gut microbiota display crucial alterations during probiotic intervention, but none of these trials have clearly stated that these alterations are solely responsible for reduction in body weight or obesity. Also, the effects of probiotic supplementation on gut microbiota modulation in context with gut permeability, satiety hormones, eCB system are needed to be studied in detail…. In addition, the comparative effect of different strains was also not studied, even though probiotic effects varied among individuals. These gaps in our current understanding open the platform for future research. Further research is required to prove their beneficial effects on humans to gain an insight into the mechanisms through which live bacterial organisms improve the human gut barrier function.

## Conclusions and Future Prospects

Obesity and related diseases have enormously increased in society and considered the biggest plausible factor for disturbing well-being and health. Studies performed in animal models, and human subjects have clearly indicated that dysbiosis of gut microbiota predisposed them toward obesity and other associated disorders. Gut microbiota influences obesity by acting on the various mechanisms that are central to energy homeostasis and development. In most of the studies, LPS stimulated low grade inflammation was understood as the prime mechanism by which gut microbiota induces obesity. Supplementation of prebiotics and probiotics addressed therapeutic effect on the altered gut microbiota that provides us an opportunity to prevent or treat obesity. However, the discrepancy observed in some studies, in the context of gut microbiota, might be due to the adoption of different sequencing techniques, intra-individual strain differences, age and genotype of individuals. In a nutshell, we believe that the science of prebiotics and probiotics have the potential to tackle obesity and associated metabolic disorders.

But before that, several problems need to be seriously addressed. Till date, it is not clear which microbial community contributes more to the obesity etiology. In some studies, a particular species was positively influenced, while contrasting results were obtained in other studies. This might be due to the complex nature of gut microbiota. The next challenge is to figure, what would be the appropriate dose of these dietary modulators for improving health. Whether they should be same for all age groups, is a major point to discuss. None of the studies have analyzed the comparative effect of different strains with regard to their anti-obesity potential, so the issue certainly requires further research. The biggest one is the safety issue of probiotics, although they are known to be safe for human consumption, but at the same time we cannot deny from the fact that they may spread antibiotic resistance (Gad et al., 2014) Likewise, some probiotics could also cause gastrointestinal disorders as previously discussed in a review (Marteau and Seksik, 2004). This reflects the need for a stricter regulatory framework globally. Products containing probiotics should be analyzed for safety risks before sale in the market.

Analyzing the crosstalk between probiotics and gut microbiota would be one of the most important future research tasks in broadening our understanding on the topic. One of the main aspects to be studied in this area would be to understand how probiotics make genetic communication with the intestinal microbiota by means of genetic material exchange. If we transform the genetic properties of probiotic bacteria in some of the intestinal bacteria, then it is possible to confer few beneficial traits to the host. There is an emerging need to look for those strategies that would not only positively modify the gut microbiota, but also be safe for use.

## Author Contributions

DD conceived the idea for the article, prepared and edited the final manuscript. R prepared the figure, tables and edited the final manuscript. MP, US, TD, NK, SK help in final editing of the manuscript. AP and PS participated in developing the idea and critically revised the manuscript. All authors approved it for publication.

## Conflict of Interest Statement

The authors declare that the research was conducted in the absence of any commercial or financial relationships that could be construed as a potential conflict of interest.
